# Clinimetrics of the 9- and 19-Item Wearing-Off Questionnaire: A Systematic Review

**DOI:** 10.1155/2018/5308491

**Published:** 2018-04-01

**Authors:** Carlos E. Mantese, Artur Schumacher-Schuh, Carlos R. M. Rieder

**Affiliations:** ^1^Postgraduate Program in Medical Sciences, Universidade Federal do Rio Grande do Sul, Porto Alegre, RS, Brazil; ^2^Mãe de Deus Hospital, Porto Alegre, RS, Brazil; ^3^Universidade Federal de Ciências da Saúde de Porto Alegre (UFCSPA), Porto Alegre, RS, Brazil; ^4^Hospital de Clínicas de Porto Alegre (HCPA), Porto Alegre, RS, Brazil

## Abstract

The treatment of Parkinson's disease (PD) with dopaminergic therapy improves functionality and quality of life. However, as the disease progresses, the wearing-off phenomenon develops, which necessitates complex posology adjustment or adjuvant therapy. This phenomenon may not be well recognized, especially if it is mild or involves nonmotor symptoms. Questionnaires were developed to improve the recognition of the wearing-off phenomenon. The questionnaires consist of a list of symptoms that patients must check if they have and if the symptoms improve with medication. A recent review by the Movement Disorder Society suggested the 19-item (WOQ-19) and 9-item (WOQ-9) questionnaires as screening tools for the wearing-off phenomenon. However, there has not been a systematic review to assess the questionnaires' clinimetric properties, such as sensitivity, specificity, test-retest reliability, and responsiveness. We conducted an extensive search for studies using these two tools. We identified 3 studies using WOQ-19 and 5 studies using WOQ-9. Both questionnaires seem to have good sensitivity (0.81–1). WOQ-19 has variable specificity (0.39–0.8), depending on the number of positive items, while WOQ-9 lacks specificity (0.1–0.69). Only one study using WOQ-19 reported test-retest, and only two studies reported responsiveness. Thus, this report describes the first independent systematic review to exam quantitatively the clinimetric properties of these two questionnaires.

## 1. Introduction

The treatment of Parkinson's disease (PD) with dopaminergic therapy improves functionality and quality of life. However, as the disease progresses, it causes motor and nonmotor fluctuations [1]. The well-described wearing-off (WO) phenomenon is the shortening effect of levodopa, which can be managed with dosage adjustment or adjuvant therapy, such as catechol-*O*-methyltransferase (COMT) inhibitors [2]. Clinical evaluation has been the gold standard for diagnosing this condition. However, the WO phenomenon may not be well recognized, mainly if it is mild or involves nonmotor symptoms. Several scholars argue that recognition of WO phenomenon could change the way that it is managed and improve patient's functionality [3].

To improve the recognition of WO phenomenon, a 32-item questionnaire (WOQ-32) was developed [3]. The questionnaire consists of a checklist of symptoms that patients must identify, and they must note if these symptoms improve with medication. For practical reasons, using the same research, this questionnaire was adapted to a 19-item questionnaire (WOQ-19), which had the same properties [4]. Later, a 9-item questionnaire (WOQ-9) was developed [5], containing the most valuable questions, and it was successfully tested [6]. The WOQ-9 has been used for a number of clinical studies, translated into several languages, and adapted with several different clinimetric properties [7]. A recent review by the Movement Disorder Society set both the WOQ-19 and WOQ-9 as recommended tools for screening for WO phenomenon [8]. However, this review did not address quantitatively the clinimetric properties in Parkinson's disease patients compared to clinical evaluation.

Thus, we conducted a systematic review and analysis of the clinimetric properties of both the WOQ-19 and WOQ-9 questionnaires, such as sensitivity, specificity, predictive positive value (PPV), negative predictive value (NPV), and stability with test-retest and responsiveness.

## 2. Methods

We follow the PRISMA statement.

The inclusion criterion was studies using WOQ-9 or WOQ-19 in PD patients to diagnose WO compared to the gold-standard, clinical evaluation. The studies must examine sensitivity and specificity, or they must include data that we could calculate. Also, we include studies using one of the questionnaires if they employed data regarding test-retest or responsiveness. Formal validation was not required [9], but at least a translation and face validation for the given language was applied. Reviews, abstracts, and conference meetings were excluded. Responsiveness was calculated following Cohen's effect size [10].

The search was conducted in MEDLINE, Embase, and Web of Sciences between 01/06/2017 and 22/12/17. The terms were ((Parkinson's OR Parkinson's disease) AND (wearing off OR wearing-off OR motor fluctuation) AND (questionnaire)). There was also a bibliography review for the select articles and reviews already published. The articles were independently selected by title for abstract reading by two reviewers (Artur Schumacher-Schuh and Carlos E. Mantese). In case of disagreement, the articles were discussed by another author (Carlos R. M. Rieder). Later, a number of articles were selected for full reading based on abstract information.

## 3. Results

**Figure 1:**
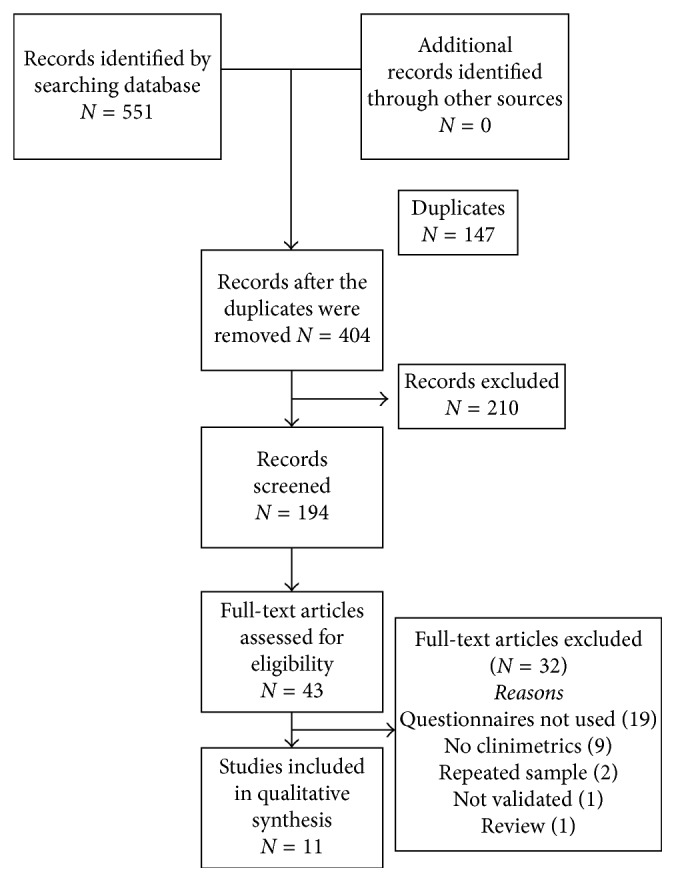
Flow diagram for data extraction.

As shown in Figure 1, we observed 404 articles based on the title after excluding duplicates. We reviewed 194 abstracts and 43 full-text articles after excluding 4 abstracts. Ultimately, we selected 3 articles about WOQ-19 [11–13] and 5 articles about WOQ-9 [6, 14–17] for sensitivity and specificity, 1 for WOQ-19 test-retest [18], 2 for responsiveness, 1 for WOQ-9 [19], and 1 for WOQ-19 [20]. Of note, we did not include the original WOQ-19 description [4] because it was an adaptation of WOQ-32, as was WOQ-9 [5]. In addition to these articles, we exclude 17 more because they did not use the target questionnaires; among these 17, two more were from the same 32-item questionnaire [3, 21] and 15 from other means of assessment [22–36]. One article was not used because it lacked any type of language validation [37], and two articles presented post hoc analyses of the same populations outlined in other articles that we include [38, 39]. Nine articles did not present clinimetric properties [40–48], and among them, two provided data using which we could calculate only PPV [41, 43]. One was a review [7]. One paper [18] included for test-retest was not included for sensitivity and specificity because it used the same population as another article [13].

We did not identify any articles that were not included in the search from original libraries.

In WOQ-19, there are 3 trials selected (Table 1). One of them used 1-item cutoff [12], while the others [11, 13] used 2-item cutoff. The sensitivity ranged from 0.81 to 0.90, and the specificity was 0.39–0.80. PPV was 0.62–0.88, and NPV was 0.64–0.84. The wide range of specificity seems secondary to one study that used 1-item cutoff. This trial does not exhibit better aggregate sensitivity but has shown worse specificity.

In WOQ-9 (Table 2), all studies had 1-item cutoff. Sensibility was 0.87–1, specificity was 0.10–0.69, PPV was 0.48–0.86, and NPV was 0.71–1. All studies showed excellent sensitivity but lacked specificity.

Test-retest stability was assessed in one paper [18], two weeks apart from each test, which showed an intraclass correlation of number of positive items of 0.858. It was applied to stable patients; however, it did not mention the clinical stability or the type of intraclass correlation.

For responsiveness, two studies were analyzed [19, 20]. Both were clinical trials with COMT inhibitors. One of them [19] used WOQ-9 and reported improvement in most items in proportion of patients with improvements; however, it did not provide data to calculate the effect size. In the other trial [20], Cohen's effect size was 0.5.

## 4. Discussion

This report describes the first systematic review of quantitative clinimetric properties of WOQ-19 and WOQ-9. Systematic reviews are fundamental to summarize important data for research and clinical practices. Additionally, this report describes the first independent review of the clinimetric properties of these questionnaires.

The WOQ-19 seems to have good accuracy, which is an excellent tool in both research and clinical practice, when a 2-item cutoff is used. However, most of the trials used WOQ-9, which has excellent sensitivity but poor specificity. Thus, the WOQ-9 could be used as a screening tool to identify certain at-risk individuals, but it would need a clinical evaluation to confirm the diagnosis, as several trials have done [42, 46]. Stacy [7] has argued that office visits could fail to recognize WO, and its position as the gold standard of care may need reevaluation [6]. This hypothesis seems difficult to prove. Moreover, most clinical trials for Parkinson's disease treatment use wearing-off outcomes diaries or UPDRS (Unified Parkinson's Disease Rating Scale) wearing-off subitems. Raciti et al. [49] showed that UPDRS has 0.87 sensitivity and 0.43 specificity compared to clinical evaluation, which make it similar to WOQ-9 and considerably worse than WOQ-19.

The variability in WOQ-19 can be explained by several reasons. In WOQ-19, one clinical trial used the 1-item cutoff and therefore lost specificity. In a ROC curve plotted by Martinez-Martin et al. [11], the questionnaire showed better accuracy when the 2-item cutoff was used. As in WOQ-9, the 1-item cutoff seems to have the same lack of specificity. Fukae et al. [17] showed that when the 2-item cutoff is used, the WOQ-9's specificity improves (from 0.39 to 0.72) and loses a little sensitivity (from 0.94 to 0.87). Additionally, each study involved different languages, and the final result depended on, in part, the properties of each specific validation. Moreover, the gold standard could be different depending on the physician's expertise (i.e., if they are movement disorder specialists or in-training neurologists). Finally, while most clinical trials excluded patients who could not complete the questionnaires, certain differences in educational, cultural, and social backgrounds could explain a portion of the variability in the questionnaires.

Of note, we did not identify information regarding questionnaire reliability or validity other than criteria validity. Furthermore, only one article on WOQ-19 examined test-retest through intraclass correlation of number of positive items, and it did not mention the type, consistency, or agreement. The second is preferred [50]. For test-retest, even not mentioned clinical stability, two weeks apart from each test seems enough time in Parkinson's disease to avoid recall bias and ensure clinical stability. We have not found any paper with kappa agreement from individual question. Being a questionnaire with dichotomous responses, the use of kappa would seem appropriate. We did not identify any reports of test-retest for WOQ-9. Responsiveness was obtained from two clinical trials for WOQ-19 comparing add-on therapy with entacapone (a COMT inhibitor). This therapy is used to treat WO phenomenon, and both showed an improvement of questionnaire on the basis of number positive items. However, in one trial, we have no data to calculate the effect size [18]. The other one [19] showed effect size of 0.5, which means a moderate effect.

The lack of data regarding reliability and even validation by means other than criteria validation might be observed because the original study used WOQ-32, and later, the WOQ-19 and WOQ-9 were developed, and even those questionnaires were not tested for those properties by the developers. Additionally, we did not include conference meetings or abstracts, which can account for the loss of certain data (even so, we did not identify those data in the libraries we searched). This information is important to clinicians and researchers because it might influence how they use questionnaires. A questionnaire with poor test-retest performance is not reliable to use, and the results can change with no change in clinical status.

We excluded several important articles, such as Stacy et al.'s [3] description of the WOQ-32 questionnaire and its transformation into the WOQ-19 [4] and WOQ-9 [5]. However, this article involves data from a different questionnaire, which was later transformed into the WOQ-19 and WOQ-9 questionnaires. Several articles were post hoc analyses of primary data, which we had already included. One study did not have any type of validation and did not meet our inclusion criteria. Most of the excluded trials were with no comparator; therefore, we could not address clinimetric properties.

## 5. Conclusions

We conducted the first systematic review of WOQ-19 and WOQ-9, an important tool for screening and diagnosing WO. The lack of certain data suggests caution when using the WOQ-9. However, the WOQ-19 exhibits reliability and was validated to use as a diagnostic tool. Moreover, we suggest that the authors report complete properties when they are publishing papers validating their methods.

## Figures and Tables

**Table 1:** WOQ-19.

Author	Year	*N*	Cutoff	Sn	Sp	PPV	NPV
Martinez-Martin et al. [11]	2008	222	2	0.88	0.8	0.88	0.79
Seki et al. [12]	2013	464	1	0.81	0.39	0.62	0.64
Stocchi et al. [13]	2014	617	2	0.90	0.63	0.76	0.84

Sn: sensitivity; Sp: specificity; PPV: positive predictive value; NPV: negative predictive value.

**Table 2:** WOQ-9.

Author	Year	*N*	Cut-off	Sn	Sp	PPV	NPV
Stacy et al. [6]	2008	216	1	0.96	0.40	0.48	0.94
Chan et al. [14]	2011	101	1	0.87	0.69	0.86	0.71
Bares et al. [15]	2012	563	1	0.98	0.27	0.72	0.91
Santos et al. [16]	2014	60	1	1	0.10	0.54	1
Fukae et al. [17]	2015	180	1	0.94	0.39	0.66	0.83

Sn: sensitivity; Sp: specificity; PPV: positive predictive value; NPV: negative predictive value.
